# Deep Learning for Reconstructing Low-Quality FTIR and Raman Spectra—A Case Study in Microplastic Analyses

**DOI:** 10.1021/acs.analchem.1c02618

**Published:** 2021-11-22

**Authors:** Josef Brandt, Karin Mattsson, Martin Hassellöv

**Affiliations:** Department of Marine Sciences, University of Gothenburg, Kristineberg 566, 45178 Fiskebäckskil, Sweden

## Abstract

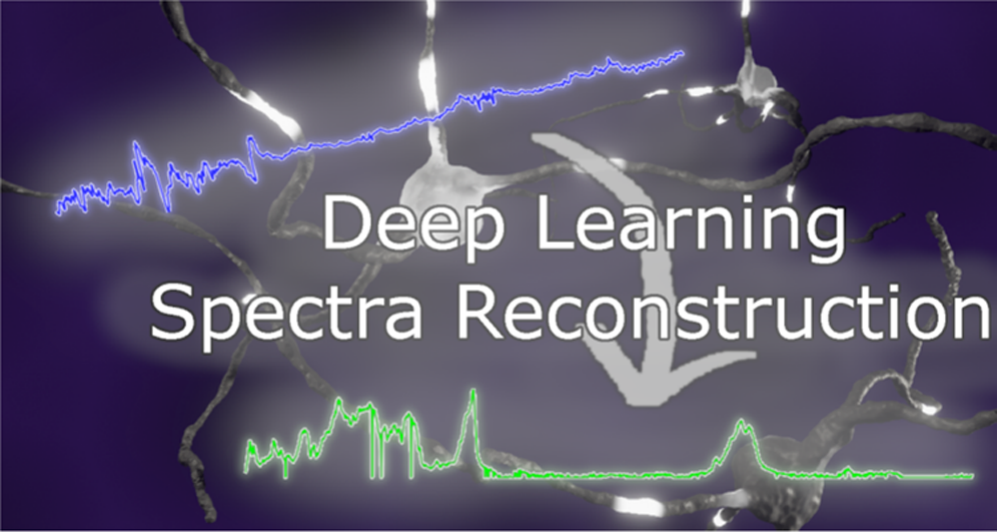

Herein we report on a deep-learning method for the removal of instrumental noise and unwanted spectral artifacts in Fourier transform infrared (FTIR) or Raman spectra, especially in automated applications in which a large number of spectra have to be acquired within limited time. Automated batch workflows allowing only a few seconds per measurement, without the possibility of manually optimizing measurement parameters, often result in challenging and heterogeneous datasets. A prominent example of this problem is the automated spectroscopic measurement of particles in environmental samples regarding their content of microplastic (MP) particles. Effective spectral identification is hampered by low signal-to-noise ratios and baseline artifacts as, again, spectral post-processing and analysis must be performed in automated measurements, without adjusting specific parameters for each spectrum. We demonstrate the application of a simple autoencoding neural net for reconstruction of complex spectral distortions, such as high levels of noise, baseline bending, interferences, or distorted bands. Once trained on appropriate data, the network is able to remove all unwanted artifacts in a single pass without the need for tuning spectra-specific parameters and with high computational efficiency. Thus, it offers great potential for monitoring applications with a large number of spectra and limited analysis time with availability of representative data from already completed experiments.

## 1 Introduction

Vibrational spectroscopy techniques are ubiquitous in polymer analytics and widely used for unknown material identification or chemical composition characterization.^1,2^ The most widely employed ones are Fourier transform infrared (FTIR) and Raman spectroscopies, both coming in a broad variety of different instruments ranging from highly sensitive laboratory instruments to portable or even handheld devices with portable convenience but accordingly weaker analytical figures of merit. Both FTIR and Raman spectroscopies are also commonly integrated in light microscopes for noninvasively studying very small specimens or features. These integrated techniques will be referred to as μFTIR and μRaman, respectively.

The best possible spectral quality is achieved by optimizing measurement-specific parameters, such as laser wavelength and energy (only Raman), spectral resolution, confocal optics, and focus (especially in case of μRaman and μFTIR). The number of scans per spectrum has a very high impact on spectral quality in both Raman and FTIR spectroscopies. Acquiring a higher number of scans or spectral accumulations increases the signal-to-noise ratio according to the famous √*n* relation, which states that with *n* accumulations, the signal intensity increases by a factor of *n*, whereas the noise only increases by a factor of √*n*. Hence, measuring longer usually translates into an increased spectral quality in terms of signal-to-noise ratio. However, also other phenomena than noise can impede spectral quality, especially in FTIR spectroscopy. Depending on the mode of acquisition, i.e., transmission, transflection, reflection, or attenuated total reflection (ATR), different artifacts can occur, such as band saturation, baseline distortions, or interferences, to only name a few.

It is, however, not always feasible to optimize all possible parameters for certain analytical tasks, especially when a high number of samples have to be processed, thus limiting the analysis time per sample. One such example is the analysis of environmental samples with respect to their content of microplastic (MP) particles.^3−5^ In such an analysis, many thousands of particles have to be scanned per sample for distinguishing MP particles from other organic or inorganic particles. As also a large number of samples need to be processed for obtaining a meaningful spatial and temporal sample coverage on relevant ecosystems, the analysis time is a highly critical parameter. The analyses are usually performed in automated measurements and large numbers of spectra are acquired that then also need to be processed in an automated fashion.^6−8^ Spectral quality is, therefore, often relatively poor, resulting in the necessity of post-processing steps for increasing the confidence in spectral evaluation. Specific phenomena can thereby be tackled with a toolbox of different methods. Noise reduction is conventionally achieved by Savitzky–Golay filtering,^9−11^ whereas baseline artifacts can be removed by different baseline-subtraction methods, such as fitting polynomials or asymmetric least-square smoothing.^12,13^ Although established, all of the mentioned methods require adjusting a variety of parameters and need to be combined to cope with different kinds of spectral artifacts simultaneously. When applied to a set of spectra containing a large variety of different spectra having different levels of noise and different kinds of baseline distortions, they will not yield ideal results for all of the spectra. Tailoring optimal post-processing settings for each spectrum is not feasible when large numbers of spectra have to be processed in little time. A given set of post-processing parameters will always work well on some spectra, but might also do more harm than good in other cases. For example, baselines are not corrected properly and additional distortions can be introduced, or finely resolved peak profiles might become highly blurred out by too intensive smoothing. Therefore, the operator needs to manually optimize the spectral processing parameters for each given set of spectra and in some cases might still not find a good solution. Finding a good solution is, furthermore, time-consuming when working on larger datasets. Many algorithms, such as baseline fittings, work iteratively and require computing many passes on the spectral data, thus requiring either dedicated hardware or long processing times.

**Figure 1:**
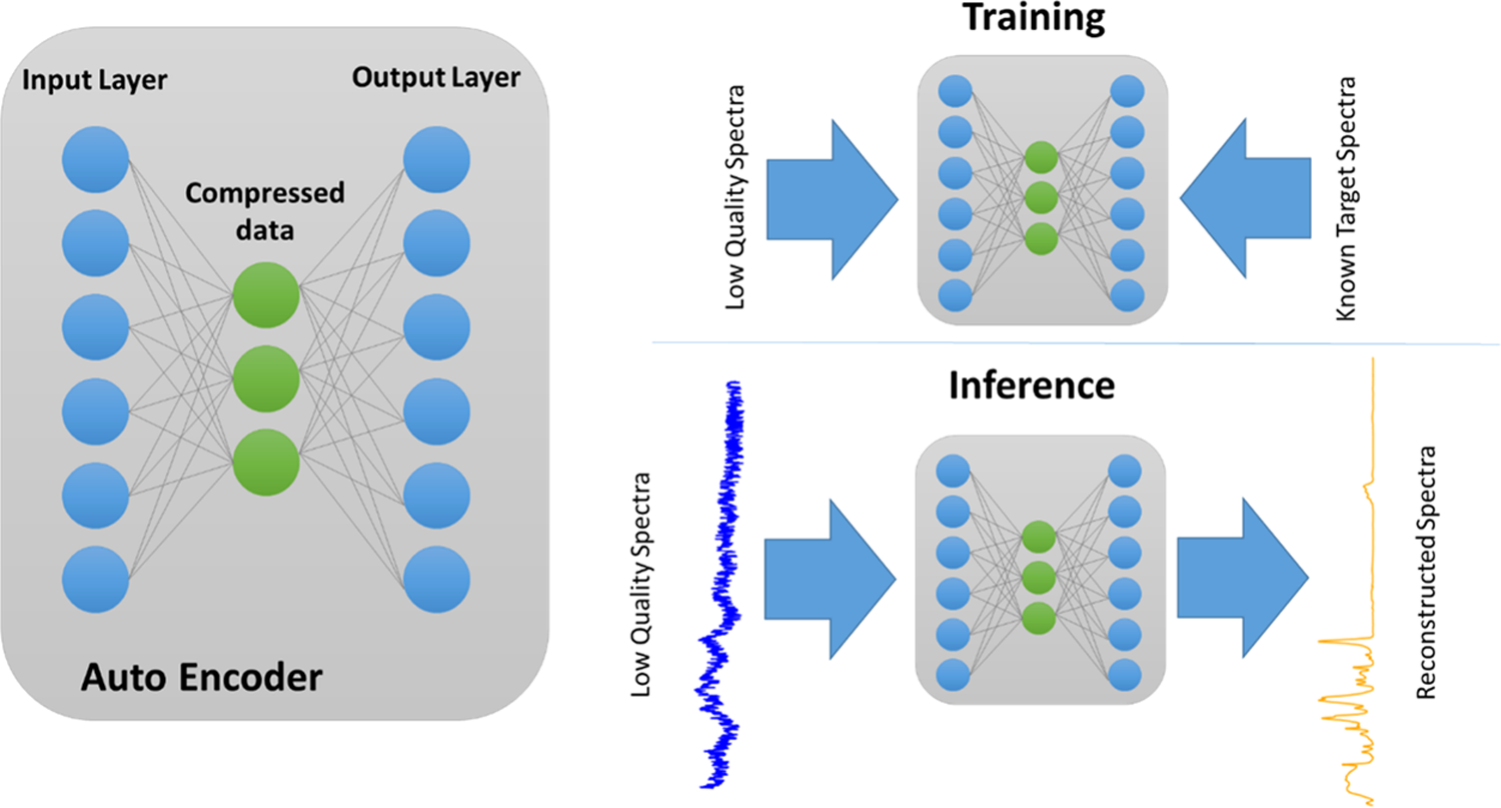
Schematic of the autoencoder network for spectral reconstruction.

Alternative methods can be found in the field of machine learning and neural networks, specifically in the form of autoencoding neural nets, in short, autoencoders. Autoencoders represent a specific architecture of neural networks comprising of two stages. First, the input data is encoded into a compressed form, using the “encoding” stage of the network (refer Figure 1). Then, the encoded data is reconstructed into the original format using the network’s decoder. In this way, the dimensionality of the input data is first reduced to only represent the essential information, which is then used for constructing the original data.^14,15^ This concept allows effectively removing noise and unwanted artifacts in vibrational spectra in a single pass, suitable to a broad variety of different spectral types.

The network is trained by passing in low-quality spectra on the one side and clean spectra on the other side. The network then learns how to encode the low-quality spectra and to expand again to recreate the unperturbed spectra. The concept was not only successfully applied in various variations in image denoising^16^ but also on hyperspectral images,^15,17^ biomedical signals,^18^ and geophysical data,^19^ and, therefore, shows great potential and can be transferred to the problem of enhancing quality of FTIR and Raman spectra with high levels of noise and/or presence of spectral artifacts. In vibrational spectroscopy, autoencoders were used for spectral classification or dimensionality reduction^20^ or training data synthesis in Raman spectroscopy^21^ and only rarely for reconstruction of spectral artifacts. Guo et al. presented the application of a one-dimensional (1D) convolutional UNet for correction of Mie scattering in FTIR spectra of poly(methyl methacrylate) (PMMA) spherules^22^ and also Raulf et al. used an autoencoder to remove artifacts from Mie scattering in FTIR biomedical images.^23,24^

The aim of the present study is to extend the application of autoencoders to the challenging domain of environmental particle analyses with both μFTIR and μRaman spectroscopies. Environmental samples are complex in composition with a broad variety of particles of different materials and shapes, being affected by different spectral artifacts simultaneously. Furthermore, we aim to explore the potential of a shallow autoencoder with only one hidden layer, which is fast and easily trained and understood, as the number of hyperparameters to tune is low. Our work is split into two parts. First, the spectral reconstruction capabilities of the neural net are assessed based on artificially distorted library spectra (both FTIR and Raman) with control over the intensity of particular spectral artifacts, which allows studying individual effects isolated from each other. Then, the method is applied to polymer spectra as obtained from microspectroscopy on cryomilled particles, possessing a variety of complex distortions simultaneously, as typically observed in the field of MP analytics.

## 2 Materials and Methods

### 2.1 Cryomilled Microplastic Particles

Poly(ethylene terephthalate) (PET) particles were generated by cryomilling pieces of a blue-tinted PET plastic bottle. The cut-out pieces of approximately 0.5 × 0.5 cm^2^ were cryomilled using a Tissue Lyser (Qiagen, Netherlands) equipped with two 10 mL stainless steel grinding jars (Retsch, Germany) in two stages. First, three cycles of cooling in liquid nitrogen (submerging the jars in liquid N_2_ for 15 min) and grinding at 30 Hz (5 min) were done with a 10 mm steel ball. After replacing the big steel ball with eight 7 mm steel balls, five additional cycles were performed with the same cooling and grinding times. The resulting particles ranged from 100 to 500 μm in size.

Poly(methyl methacrylate) (PMMA) particles were obtained after cryomilling pristine preproduction pellets of PMMA using the same procedure as for the PET. The obtained particle sizes ranged from 20 to 200 μm.

Particles of polypropylene (PP), polystyrene (PS), and polyethylene (PE) were obtained from plastic objects, such as drinking bottles, canisters, or screw caps, from beach litter surveys at the Swedish west coast. The particles were obtained by filing the objects with a metal file, resulting in particles in the range from 20 to 300 μm.

Particles of poly(vinyl chloride) (PVC) were supplied by Carat GmbH (Germany) and were delivered in a size fraction of 100–300 μm, and the original material, a raw compound material with additives, was acquired from a European Compounder specialized in PVC.

### 2.2 μFTIR Measurements

Particle analyses were conducted on a Nicolet iN10 MX Infrared Microscope (Thermo Fisher). About 1500 particles were deposited on a metal-coated microscope slide for particle measurement. Using the self-developed measurement and analysis software GEPARD,^7^ an optical image was acquired using the external side illumination (resembling dark-field illumination), which was used for automated particle recognition. For each recognized particle, a rectangular FTIR aperture was calculated such that the aperture optimally covers the particle without exceeding its boundary. A maximum aperture size of 150 × 150 μm^2^ was set to avoid saturated spectra on large particles. For background spectral acquisition, the needed apertures were grouped using a 10% area margin, and for each group, a background spectrum with a square aperture representing the group aperture area was acquired at an empty spot on the microscopy slide. After acquisition of all background apertures, the stage was driven to each particle location and a spectrum was acquired. Background and sample measurements were conducted in reflection mode at a resolution of 4 cm^–1^ and 32 scans per acquisition. The sample spectra were background corrected by calculating −log 10(sample spectrum/background spectrum).

### 2.3 μRaman Measurements

Spectra from different known MP particles such as pristine preproduction pellets and reference particles from the JPI Oceans project BASEMAN were obtained on a Raman microscope (Alpha 300, WITec, Germany) equipped with a 532 nm laser and a 600 l/mm spectroscopic grating. The particles were measured on metal-coated membranes at 20× magnification with 50 accumulations at 0.5 s acquisition time, where the laser power and magnification were optimized manually for each particle. The used software for acquisition was WITec Control FIVE.

### 2.4 ATR Database Spectra

A set of 174 clean ATR reference spectra of different polymers, as well as organic and inorganic compounds that are frequently found in microplastic analyses, was obtained with permission from simple-plastics.eu/download.html. The database was first described by Primpke et al.^25^

### 2.5 Data Processing

All data handling was done in Python (3.8), and the code is available at https://github.com/Brandt-J/SpectraReconstruction. Using standard numeric calculation libraries, such as numpy (1.20.2), scikit-learn (0.23.2), and scipy (1.6.2), a set of functions was created to artificially distort the clean ATR FTIR spectra; the functions can be found in the repository in the distort.py script file. Specifically, functions were built to add random noise, baseline bending, ghost peaks, fluorescence contributions, and cosmic-ray peaks. For understanding how these functions are used to create the herein-described experiments, the repository includes a folder entitled “ManusciptImages” containing the script files that reproduce the herein-shown figures. These scripts contain all details about the exact experimental setup and training parameters.

### 2.6 Neural Net Architecture

The following model comprising an encoder and a decoder was created using Tensorflow (2.3.0) and Keras (2.4.0) in Python (3.8); the code is also included in the online repository. The encoder is a densely connected sequential network with one layer mapping the input spectrum from 1024 down to 128 latent dimensions. The encoder expands to 1024 output dimensions again for restoring the clean spectra. If strong overfitting was observed, additional dropout layers were introduced after the input and the hidden latent layer, with a dropout of 0.15 each. For testing, also a one-dimensional convolutional network was created, and the exact architecture can be seen at https://github.com/Brandt-J/SpectraReconstruction/blob/main/Reconstruction.py.

All spectra that were fed into the network were mapped to a wavenumber axis having 1024 wavenumbers and were normalized to a 0.0–1.0 range.

### 2.7 Neural Net Training and Evaluation of Reconstruction Quality

This requires working with two similar, yet not identical, datasets for training and evaluation, i.e., the training and testing dataset. It is important not to test the reconstruction performance directly with the data the network was trained with. Machine-learning models are inherently prone to overfitting, which means that the model “remembers” very specific characteristics of the training dataset but does not generalize well, and performs worse when applied to previously unseen data. Using a second dataset that the model has not seen during training allows spotting such an overfitting behavior. The reconstruction quality is obtained by comparing all reconstructed spectra with the expected spectra. The Pearson correlation coefficient is computed for each spectral pair and the corresponding correlation distribution serves as a measure for the reconstruction efficiency.

### 2.8 Used Hardware

All computations were performed on a Dell Latitude 7400 notebook having 32 GB RAM and an Intel Core i7 8665U 4-core CPU operating at 1.90 GHz, running under Windows 10 (64 bit).

## 3 Results and Discussion

In the following section, we describe the application of the autoencoding network to different cases in vibrational spectroscopy and outline the possibilities and limitations of our method. The first three sections are devoted to benchmarks of the autoencoder on synthetically distorted spectra. Training and evaluating the autoencoder requires a large number of spectra that are beset with different spectral artifacts to a degree where they are hardly recognizable anymore. However, certainty about the ideal appearance of each spectrum is required for both training and evaluation, which makes usage of synthetic data for the first stages of the method development preferable. The fourth section then extends to real microspectroscopy measurements, being representative for measurements of microplastic particles.

### 3.1 Noise Reduction

**Figure 2:**
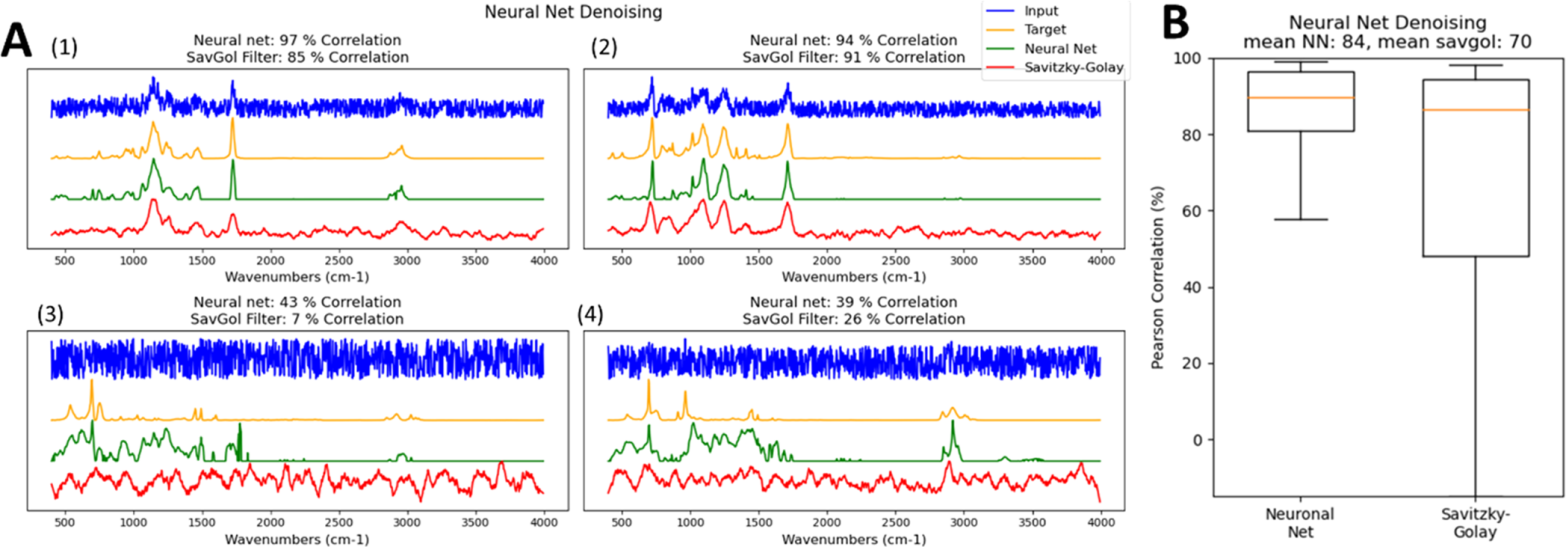
Noise-Reduction capacity of the autoencoder when compared to Savitzky–Golay filtering. (A) Four examples of denoising: blue: noisy input; yellow: expected clean spectrum; green: reconstruction from neural net; and red: spectrum smoothed by Savitzky–Golay filter. (B) Distribution of correlation of reconstructed spectra to expected spectra, as from all 4000 reconstructed test spectra.

At first, the autoencoder is used for reduction in noise, a critical aspect when working with short integration times and low numbers of accumulations. For the first assessment, the neural net was trained on 90 ATR FTIR spectra, each with 500 variations of added random noise (45 000 training spectra in total). Figure 2 depicts four examples from the validation run, where the net was used for restoring 20 other ATR FTIR spectra not included in the training session with 200 variations of added random noise each (4000 testing spectra in total). The four panels in Figure 2A compare noise-reduction capabilities of the neural net as compared to a Savitzky–Golay filter (window length: 15 pts, first-order polynomial, see Figure S1 for a benchmark of Savitzky–Golay parameters). Figure 2B shows the complete statistics of the achieved reconstruction correlation coefficients as obtained from the neural net and the Savitzky–Golay filter. Panels A(1) and A(2) show the potential of the autoencoder in restoring the original spectral shape, even from spectra beset with very high levels of noise. The experiment shows that even sharp band features can be restored correctly without having them smoothed out, as conventional noise-reduction methods typically do. On the contrary, panels A(3) and A(4) also clearly illustrate the autoencoder limitations. When a conventional smoother such as the Savitzky–Golay filter performs poorly (i.e., the correlation to the actually desired spectrum is low), the smoothed spectrum is itself still noisy and not clearly recognizable as a spectrum at all. On the other hand, the autoencoder produces clean spectra of high signal-to-noise ratios in all cases. Low correlation to the desired spectrum is not a result of poor quality of the produced spectrum but rather of wrong peak positions, shapes, and intensities. In other words, when only being presented with the corrected spectra, identifying “poor” results from a conventional smoother is straightforward. In contrast, it may be more difficult to identify incorrectly reconstructed spectra produced from the neural net. Figure 2B, on the other hand, shows that in the present exercise of reconstructing the 4000 test spectra, the neural net produced spectra that showed higher correlation to the target spectra than the Savitzky–Golay filter.

### 3.2 Removal of Complex Spectral Distortions

**Figure 3:**
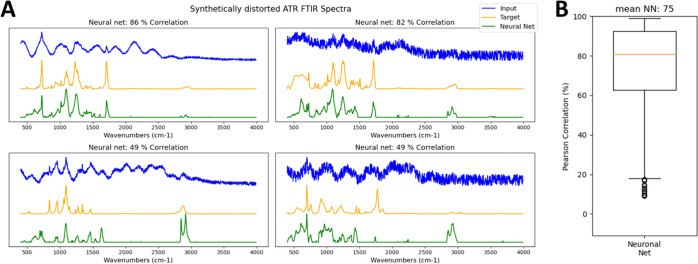
Restoration of original spectra from noise and spectral distortions simultaneously using the autoencoder. (A) Application to synthetically distorted ATR spectra and (B) correlation distribution of the neural net reconstruction.

It is important to note that the autoencoder is not *per se* a structure for only performing denoising, but that it can be used to reduce very different spectral artifacts, such as from refraction, scattering, partial transmission/reflection, or saturation altogether. To assess the general spectral reconstruction capabilities, the neural net was trained with 60 different ATR reference spectra, each with 100 variations of random noise and baseline distortions. Then, a test was run on 40 other ATR reference spectra with 100 variations of random noise and distortions each. Figure 3A shows four examples of different reconstruction qualities of the neural net, whereas Figure 3B summarizes the reconstruction quality in terms of correlation to the target spectra of all 4000 test spectra. Again, the neural net performs very well in the majority of the cases to restore the original spectra from the added random noise and distortions. It only fails in very difficult situations where even by close human inspection the original bands are not visible. Removing such a broad range of spectral artifacts by a combination of noise filtering and baseline subtraction would be a highly challenging task.

As an additional benefit, the application of the trained neural net to the test data is computationally very efficient and runs extremely fast: For example, restoring 40 000 spectra with 1024 wavenumbers each takes only about 0.2 s on a standard office notebook PC (no dedicated GPU needed).

### 3.3 Removal of Typical Raman Artifacts

The previous section was related to the removal of artifacts in FTIR spectra. The autoencoder can be similarly used for post-processing of Raman spectra. In Raman spectra, the most critical issues typically arise from low signal-to-noise ratio, dominant fluorescence contribution, and cosmic-ray peaks. For some particle types, interferences can occur, adding periodic baseline artifacts that can be mistaken for broad peaks by some algorithms.

The effective removal of noise by the autoencoder was already demonstrated in Section 3.1 and shall not be further discussed again. Both fluorescence and cosmic-ray removal are less difficult to remove as they are either significantly broader (fluorescence) or narrower (cosmic rays) than typical Raman bands. Noise reduction in Raman spectra is slightly more challenging as in FTIR spectra. As Raman bands are typically narrower, the risk of smoothing out band profiles by too aggressive noise suppression increases. Furthermore, Raman detectors (charge-coupled device, CCD, sensors) usually decrease in sensitivity at higher wavenumbers, which is especially true when irradiating with high wavelength laser light, such as 785 nm. As a consequence, the signal-to-noise ratio increases with increasing wavenumbers and bands at wavenumbers higher than 2000 cm^–1^ can become very weak.^26^

Fluorescence, noise, and cosmic-ray peaks can be removed with conventional algorithms,^12,27,28^ which however also rely on adjusting fitting parameters and thresholds and require tailoring to the given spectral characteristics to perform best. Removing the periodic baseline distortions is more difficult by conventional baseline removal techniques as they can be mistaken as broad Raman bands.

**Figure 4:**
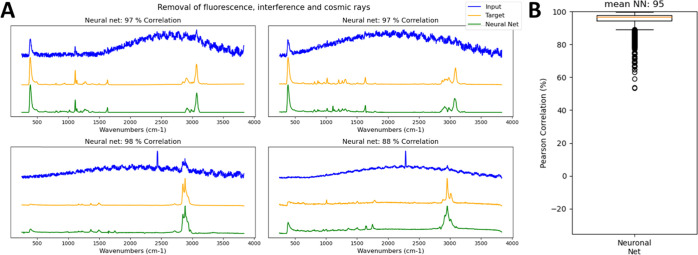
(A) Removal of artificial fluorescence and cosmic rays from Raman spectra (based on 23 different spectral types with 1000 random distortions each). (B) Boxplot of Pearson’s correlation of the reconstructed to the target spectrum.

A practical assessment based on artifact-free Raman spectra was performed to reproduce challenging scenarios from Raman microspectroscopy. Twenty-one clean μRaman spectra of different polymer particles were each modified in 1000 variations with different levels of generated noise (with increasing noise levels at high wavenumbers), cosmic-ray spikes, and baseline interferences. 80% of these spectra were used for training and 20% for validation. Figure 4 shows that the autoencoder removes all unwanted artifacts while retaining the sharp Raman band profiles.

### 3.4 Removal of Complex Distortions in μFTIR Spectra

Acquiring reflectance μFTIR spectra of small particles can lead to a complex mixture of all previously described distortions. High noise levels can occur due to short acquisition times or imprecise focusing, band profiles can be altered due to Mie scattering (leading to partially inverted bands), and/or detector saturation and background contributions can occur because of drifts in atmospheric composition and nonideal background acquisition routines. For time reasons, it is not common to acquire an individual background spectrum for each sample spectrum.

**Figure 5:**
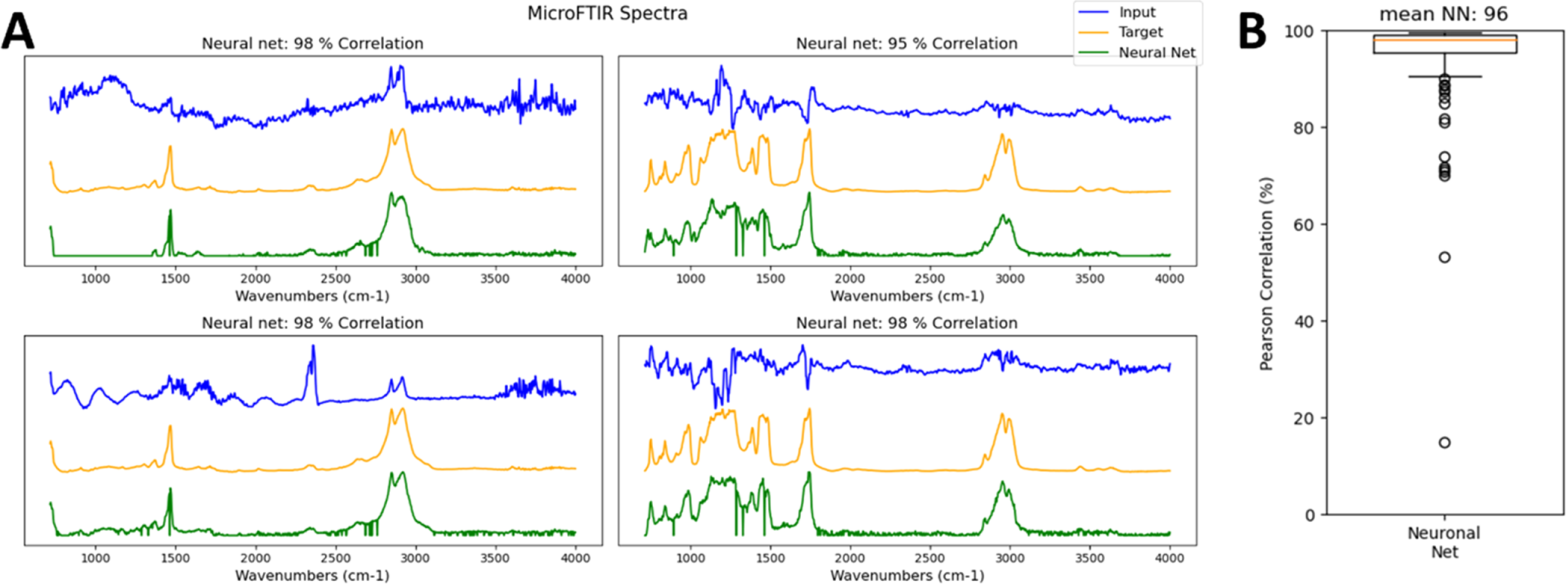
Restoration of FTIR of complex spectral distortions including Mie scattering. (A) Application to real μFTIR spectra of cryomilled particles and (B) the corresponding correlation distribution.

Figure 5A, B shows the results of spectral reconstruction applied to a dataset of μFTIR spectra of cryomilled plastic particles acquired in reflectance (or, more precisely, transflection) showing combinations of the described spectral artifacts.

The original dataset consisted of about 4500 particle spectra, from which only spectra with a correlation to the target spectrum of less than 0.5 were used, resulting in about 450 spectra that were randomly split 50/50 into training and testing data. The resulting mean correlation of reconstructed to the target spectra of 96% indicates almost perfect restoration of the test spectra. Removing such a broad variety of spectral artifacts would not be possible by any other conventional technique.

### 3.5 Potential and Limitations of the Neural Net

Our results demonstrated the unprecedented capacity of the neural net to recognize and restore any kind of spectral artifacts that hinder identification through database searches, far surpassing any conventionally applied combinations of noise filtering and baseline subtraction. All disturbing spectral artifacts can be removed in a single pass and, given the high computational efficiency of neural nets, in very short time. Conventional techniques can be used to tackle specific artifact types, as outlined in the previous section, but require precise tuning of their respective parameter sets, which might work well for individual spectra, but is virtually impossible in batch processing. The proposed neural net method shows a high potential for batch processing spectral sets showing a variety of different artifacts, such as noise or different kinds of baseline distortions. A prominent example for such batch processing cases is the analysis of environmental samples regarding their MP content. Acquiring spectra from heterogeneous samples with limited time per spectrum results in challenging spectral sets. Similarly, also the time available for spectral evaluation is limited, rendering the individual optimization of processing parameters to each spectrum virtually impossible.

However, there is one critical caveat in using the autoencoder. In contrast to conventional noise filters and baseline-subtraction methods, a neural net needs *a priori* knowledge and has to be trained on a representative set of training spectra. Also, when applying the neural net to new spectra, it cannot be tuned by adjusting parameters such as the window size or degree of polynomial fitting in conventional algorithms. Changing its behavior is only possible by retraining with more or better fitting data (where transfer learning is of course an option^29^) and/or changing the network architecture. To illustrate the issue of improper training, Figure S2 in the Supporting Information shows the outcome when applying the network to the μFTIR particle spectra after training on synthetically distorted ATR spectra, which is not a suitable training set. The mean correlation of restored to the target spectra suddenly drops to only 29% (of previously 96%). The phenomenon of a model to work well on training but poorly on validation data is referred to as overfitting and can be reduced by adding regularization^30^ and/or dropout^31^ to the used neural layers. An example of the effect of using dropout layers to reduce overfitting is illustrated in Figure S3 in the Supporting Information. Still, using appropriate training data is definitely the preferable option.

**Figure 6:**
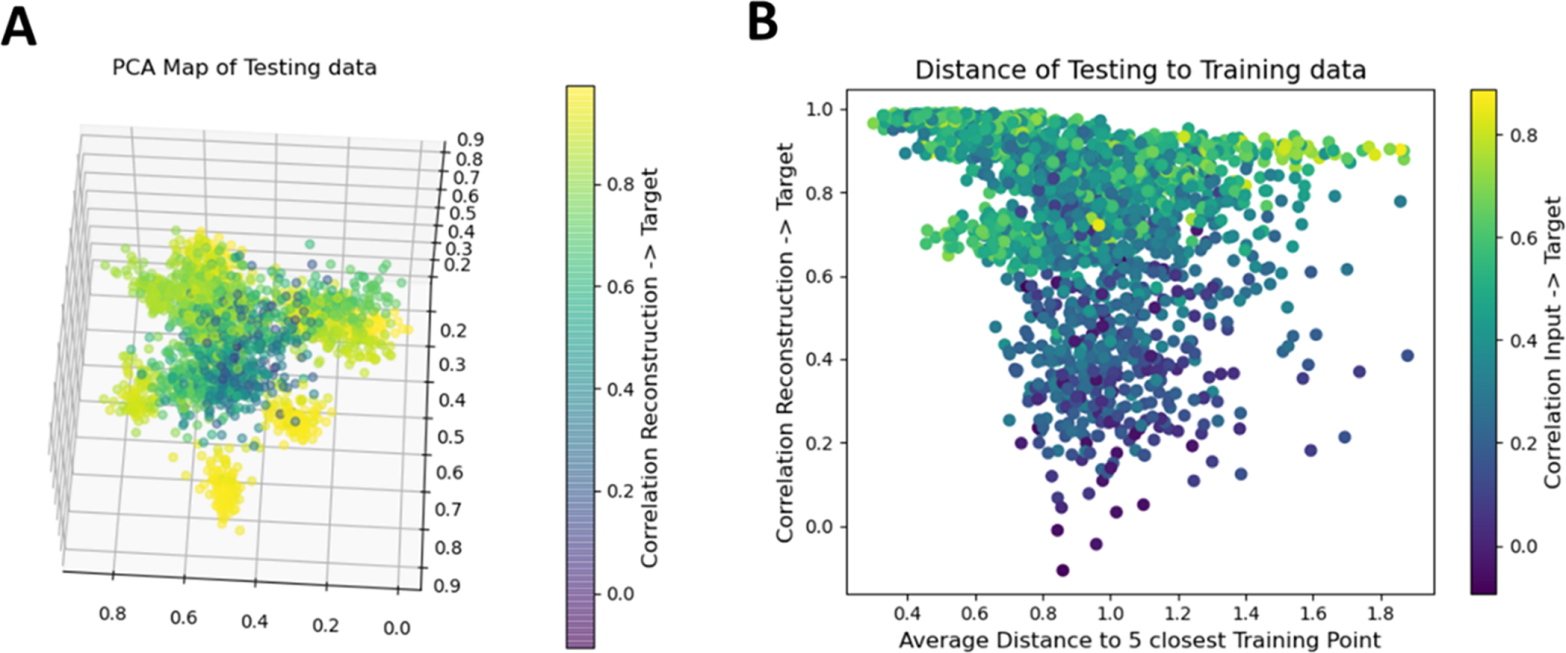
(A) Three-dimensional (3D) PCA plot of the validation with encoded quality of reconstruction (yellow = high correlation to the target spectrum, blue = low correlation to the target spectrum). (B) Plot of correlation of the restored to the target spectrum as a function of distance of each test point in the encoded space to the encoded versions of the training data.

One further critical point in the application of the neural net is how to detect faulty reconstructions. As stated in Section 3.1, detecting a wrongly reconstructed spectrum is more difficult than for conventional smoothing or correction techniques, as the network will always create a clean-looking spectrum, just probably not with the correct bands at the correct positions. Having any measure of confidence about the network’s reconstruction would be very useful for rejecting faulty reconstructions. A solution to this could be found within the encoded data layer of the network itself. Figure 6A shows a visualization of the first three principal components of the encoded version of 2000 artificially distorted ATR spectra (i.e., the testing set) that were encoded by the network after training on 8000 other artificially distorted ATR spectra (i.e., the training set). To obtain the plot, PCA decomposition was carried out on a combined array from encoded train and test data, i.e., a 10 000 × 128 matrix. The first three principal components of the test data are plotted and color-coded according to the correlation of the reconstructed spectra to the target spectra. The image shows that spectra resulting in low reconstruction quality are represented by a diffuse point cloud in the center surrounded by clusters of points representing spectra that could be restored with high quality. Hence, there is a correlation between spectra in their encoded form and the quality of reconstruction. This relation was explored further by calculating the average distance to the five closest training spectra in the encoded space for each encoded testing spectrum. Figure 6B plots the achieved correlation from the reconstructed to the target spectrum as a function of the average distance to the training data. In addition, the plotted data points are color-coded using the correlation of the input spectra (i.e., the artificially distorted spectra) to the target spectra. The image shows two trends: First, the reconstruction quality in terms of correlation of the reconstructed to the target spectrum decreases with increasing distance to the training data, which again underlines the necessity for training data as representative as possible. But, second, even for input spectra with high distance to training data, the reconstruction can be good if the input spectrum was already quite close to the target spectrum.

The code developed for this manuscript implements a simple method for calculating the average distance of any spectrum in inference mode to all of the spectra used for training the network. This allows determining the indices of spectra further away from any training data than a given user-defined threshold, which then can be used to exclude reconstructions with low confidence. However, the method is far from ideal, as the correlation between training point distance and reconstruction quality is relatively weak.

### 3.6 Potential Use Cases and Method Development

The neural net in its current form can be a very good solution for specific monitoring applications such as process monitoring or also environmental monitoring of specific sites. There, the number of possible spectral types that can occur is limited and, more importantly, known from previous experiments. The network can be trained on previously processed and categorized data and then be applied to new spectra from similar experiments. One potential application is the herein-described analysis of environmental samples regarding their content of MP particles. Other applications could be real-time spectroscopy coupled with separation techniques such as size exclusion chromatography (SEC). FTIR or NMR (Nuclear Magnetic Resonance) detectors are only rarely used in conjunction as the high levels of dilution and the short measurement times per spectrum usually result in very challenging spectral evaluation.^32,33^

When a potential use case is identified, it is critical to have training data comprising as many different spectral types as possible, each with as many variations as possible of occurring spectral artifacts. Considering the herein-used case of MP analytics with μFTIR and μRaman, a suitable training set would contain as many as possible relevant plastic spectra and also a good variety of spectra from environmental particles. Ideally, the trained neural net is then applied to measurements using the same instrument and same or at least similar settings as used for creating the training data. Setting up appropriate datasets is facilitated using measurement tools that store all measurement-relevant information, such as spectra, measurement conditions, and assignments in easily exploitable data formats, such as the open source GEPARD tool for Raman and FTIR particle measurements.^7^

The number of training data can be artificially increased using data augmentation techniques such as synthetic minority oversampling technique (SMOTE)^34^ or specific generative neural net architectures. Generative adversarial networks (GANs), for instance, are well known for their capability to produce random samples with defined features and could be used to synthetically realistically relevant spectra in a large number. Houston et al. used a similar technique (based on an autoencoder, rather than a GAN) in their study,^21^ and a simple example case for a GAN for spectral synthesis is included in this study’s code repository. Figure S4 in the Supporting Information shows how the GAN can generate polystyrene and polypropylene spectra presenting a typical μFTIR spectral appearance. Data augmentation can be very useful, especially when considering that a more densely populated training space allows for more reliable spectral reconstruction (Figure 5).

A very simple autoencoder architecture with only one hidden dense layer was used for the presented use cases. It showed good performance for the respective tasks and, depending on the size of the training set, took only 30 s up to a few minutes for training, thus allowing quick and easy iteration on data preprocessing. In addition, a one-dimensional convolutional network was tested but did not show increased performance despite significantly longer training times. Adapting and optimizing the network structure can be automated in Python using the keras-tuner package, which allows procedural generation of models within a defined range of parameters, while automatically testing and monitoring the performance of each generated model. These tools allow tailoring the autoencoder approach to other use cases.

### 3.7 Outlook

The presented study can merely act as a starting point in exploring the potential of neural nets for processing challenging spectra. The demonstrated benefits should be a high motivation to further explore the use of neural nets in processing low-quality vibrational spectra. Many different flavors of autoencoding networks have been presented in the literature, each being tailored to perform particular tasks. Gogna et al. reported on a “label-consistent” autoencoder architecture for biomedical signal reconstruction that not only reconstructs signals but also simultaneously performs classification,^18^ whereas Zhao et al. present the “what–where” autoencoder that performs especially well on large numbers of unlabeled data.^35^ Dong et al. published a comprehensive review about the different autoencoder architectures and their characteristics in general.^36^

One related task would be the capability of a neural net in directly classifying the spectra. The high capacity of deep neural nets can be used for very complex classification tasks, as can be seen in the field of image processing. New proposed neural net architectures are often benchmarked on publicly available datasets, such as the imagenet dataset, containing more than 1 million images of more than 1000 different classes.^37^ The ResNet50 is a commonly used network architecture that can be trained to such huge datasets, resulting in a very powerful network with an excellent generalizability potential.^38,39^ For the effective development of automated spectra processing routines, there is a great need for publicly available, realistic, and comprehensive test datasets that can be used for benchmarking. It is otherwise difficult to truly compare the performance and robustness of different data-processing strategies.

## 4 Conclusions

Herein, we showed the potential of autoencoding networks to reconstruct FTIR spectra from spectra that had to be acquired on unoptimized conditions and, therefore, are characterized by high levels of noise and different kinds of baseline and peakshape artifacts simultaneously. The method is of high relevance for monitoring approaches where the expected type of spectra and distortions is known and can be extracted from previous experiments. Having a specifically trained network allows for a very robust and fast restoration of the spectra, far beyond what is possible with combinations of conventional techniques, such as Savitzky–Golay noise filtering, baseline removal, or peak deconvolution/fitting. Furthermore, the low inference times of the network make the method suitable for batch processing with high numbers of spectra to work on. Data augmentation techniques, such as using generative adversarial networks, can help providing sufficient training data.

The most critical aspect is to find a reliable measure for the reconstruction confidence. The herein-proposed correlation between the distance of inference spectrum to training spectra in the latent space gives some first indications, but further investigations are needed for finding robust measures. Our work aims to motivate other researchers to explore the potential of neural nets in the field of vibrational spectroscopy and also points out to the necessity of standardized benchmark spectral sets that can be used for assessing the effective performance of any spectral processing approach based on comprehensive and realistic data.

## Supporting Information Available

Indepth investigations of the autoencoder spectral reconstruction (PDF)
Zip file containing all used data and Python code (ZIP)
