# Supplementary material for: Deep Learning for Reconstructing Low-Quality FTIR and Raman Spectra—A Case Study in Microplastic Analyses
Source: Anal Chem. 2021 Nov 22;93(49):16360–8. doi: 10.1021/acs.analchem.1c02618 (PMC8674871; doi:10.1021/acs.analchem.1c02618)
Supplement: Supplementary file 1 — ac1c02618_si_001.pdf [file ac1c02618_si_001.pdf]

--SUPPORTING INFORMATION --

Deep Learning for reconstructing low quality FTIR and Raman spectra – a case study in microplastic analyses

Josef Brandt*[1], Karin Mattsson[1], Martin Hassellöv*[1]

josefbrandt@gu.se, martin.hassellov@gu.se

[1]University of Gothenburg, Department of Marine Sciences, Kristineberg 566, 45178 Fiskebäckskil, Sweden

Abstract:

This document contains graphics/figures for a deeper understanding of different aspects of auto-encoder training and application.

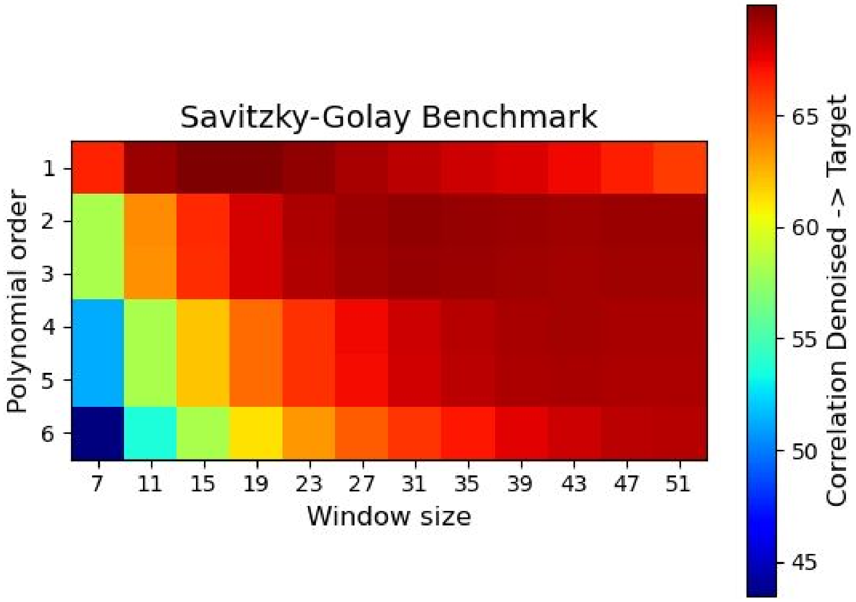

Figure S 1: Benchmark of Savitzky-Golay smoothing performance. The test was run on the same 4,000 spectra that are basis of the experiment in Figure 2 in the main manuscript. Maximum correlation of these spectra to the clean target spectra is highest with window-length 15 and polynomial order 1 (70 % correlation).

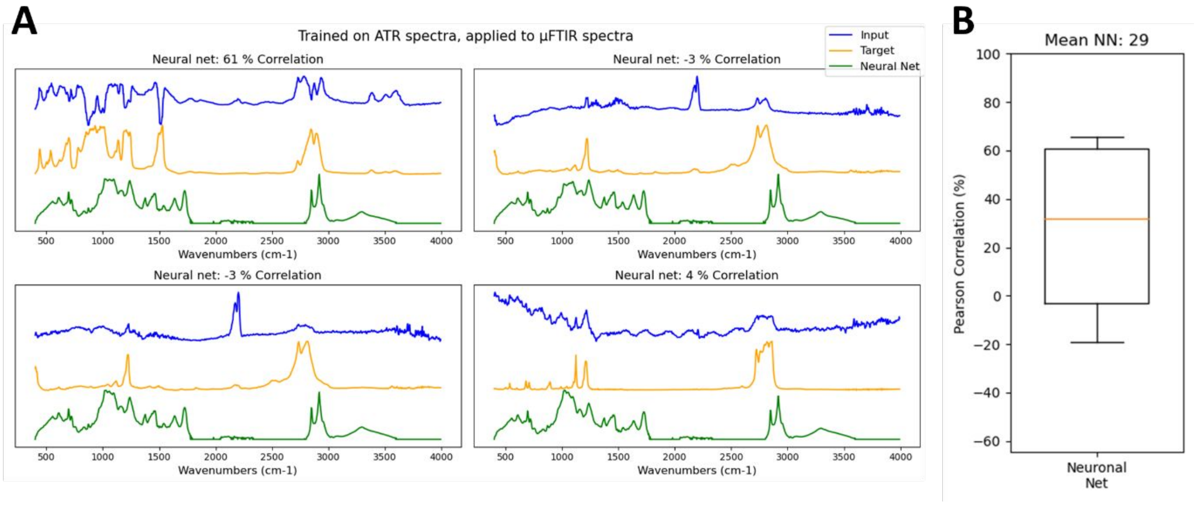

Figure S 2: Spectra examples (A) and correlation distribution (B) when trying to restore µFTIR spectra with synthetically distorted ATR spectra, which is not a suitable training set for these spectra.

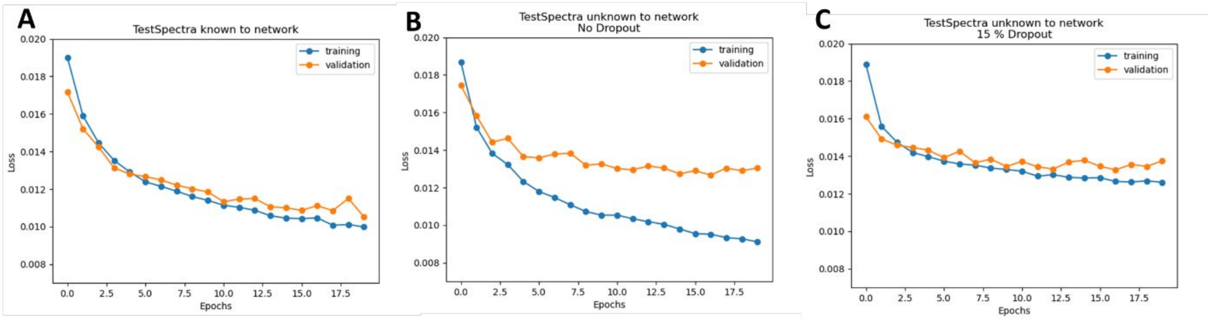

Figure S 3: Evolution of loss for training and validation with different overfitting scenarios. Ideally, the loss should reach 0.0 for perfect spectrum restoration. The spectra were generated from 100 ATR spectra with 100 variations each. 60 % of the data was used for training, 40 % for validation.

A) Test and Training sets were created from the same set of spectra (all 100 spectra). They only differ in the randomly added noise and distortions. **B)** Same architecture as in A), but test spectra are based on spectra that were NOT used in training (60 spectra types * 100 variations for training, 40 other types * 100 variations for validation). There is a significant gap between training and validation (i.e., test) loss. **C)** Same train and test data as in B), but with added dropout layers (15 % dropout) for minimizing the overfitting. Note, that the gap between training and validation substantially decreases, although the loss of the validation data remains essentially constant. However, having training and validation close together resembles better generalization performance of the network, allowing for a more realistic estimation of performance on unseen data.

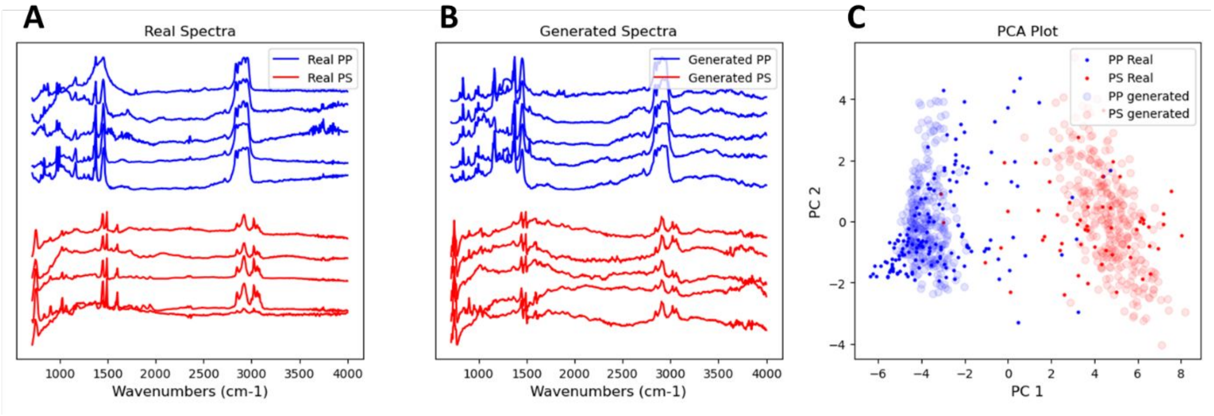

Figure S 4: Real µFTIR Polystyrene and Polypropylene spectra used for training a Generative Adversarial Network (A). Examples of artificially generated spectra after training the network for 1000 epochs (B) and a PCA plot showing homogeneous distribution of the generated spectra (C).
